# The Instrument for Measuring the Implementation Situation of Traditional Chinese Medicine Guideline: Evaluation and Application

**DOI:** 10.1155/2017/2861924

**Published:** 2017-10-26

**Authors:** Hui Li, Yangyang Wang, Yaolong Chen, Xiaoyun Wang, Chuanjian Lu, Jingwen Deng

**Affiliations:** ^1^Department of Standardization of Chinese Medicine, The Second Affiliated Hospital of Guangzhou University of Chinese Medicine, Guangzhou, Guangdong, China; ^2^Department of Standardization of Traditional Chinese Medicine, Guangdong Provincial Hospital of Chinese Medicine, Guangzhou, Guangdong, China; ^3^Engineering and Technology Research Center of Standardization of Traditional Chinese Medicine, Guangzhou, Guangdong, China; ^4^School of Medicine, Lanzhou University, Lanzhou, Gansu, China; ^5^Department of Gynaecology, The Second Affiliated Hospital of Guangzhou University of Chinese Medicine, Guangzhou, Guangdong, China; ^6^Department of Gynaecology, Guangdong Provincial Hospital of Chinese Medicine, Guangzhou, Guangdong, China

## Abstract

Clinical practice guidelines play an important role in reducing the variations in clinical practices and improving the quality of care. To assess the real effect, measuring its implementation situation is needed. The implementation situation can be reflected by testing the consistency between the actual clinical practice and the guideline. We constructed an instrument to measure the implementation situation of Traditional Chinese Medicine (TCM) guideline through consistency testing. The main objectives of our study were to validate the instrument and evaluate the implementation situation of menopause syndrome guideline of TCM, using the data from the consistency test of comparing the medical records with the guideline. A total of 621 cases were included for data analysis. Cronbach's Alpha coefficient is 0.73. The model fit of 7 items in four dimensions was good (SRMR = 0.04; GFI = 0.97; NFI = 0.97; TLI = 0.96; CFI = 0.98; AGFI = 0.90). This instrument is of good reliability and validity. It can help the guideline developers to measure the implementation situation, find the reasons affecting the implementation, and revise the guideline. The method of using consistency test to measure the implementation situation may provide a sample for evaluating the guideline implementation in other fields.

## 1. Introduction

Clinical practice guidelines (CPGs) are defined by the Institute of Medicine as “statements that include recommendations intended to optimize patient care that are informed by a systematic review of evidence and an assessment of the benefits and harms of alternative care options” [1]. CPGs have been identified as an important instrument to reduce the practice variations and improve the quality of care of patients [2–5]. A lot of resources have been used in its development and implementation [6–8]. The development standards and checklists of CPGs have been developed by World Health Organization (WHO) [9], Guidelines International Network (G-I-N) [10], Institute of Medicine (IOM) [1], Scottish Intercollegiate Guidelines Network (SIGN) [11], and National Institute for Health and Care Excellence (NICE) [12]. And in the field of guideline implementation, a lot of tools like GuideLine Implementability Appraisal (GLIA) [13], Guideline Implementability Research and Application Network (GIRANet) [14], Implementability Framework and Guideline Implementability for Decision Excellence Model (GUIDE-M) [15], and other strategies of guideline implementation [6, 16, 17] have been developed to support the implementation of CPGs. A successful implementation is an important way to achieve the maximum efficiency of guidelines. The above tools mainly focus on how to promote the implementation of guideline. However, how to evaluate the implementation situation of guideline was not mentioned.

Evaluating the implementation situation can help guideline developers to know the real implementation situation and room for improvement of the guideline. It would be conducive to making an accurate assessment on implementation effect.

The implementation situation can be reflected by consistency test, which is a method to test the consistency degree by comparing the real diagnosis and treatment practices with the guideline. In our research, we planned to use consistency test to monitor the implementation situation of guideline of Traditional Chinese Medicine (TCM). Based on the definition of consistency test, we developed an instrument to measure the implementation situation of TCM guideline. The main purposes of our study are to use climacteric syndrome guideline of TCM [18] as an example to evaluate the reliability and validity of the instrument, and use it to assess the guideline's implementation situation by testing the consistency degree between the actual clinical practice and the menopause syndrome guideline of TCM.

## 2. Materials and Methods

### 2.1. Construction of the Instrument

We reviewed the framework of 115 clinical practice guidelines of TCM. The subjects of these guidelines are related to internal surgery, tumor, gynecology, pediatrics, and dermatology. This review identified 11 items (disease diagnosis of TCM, diagnosis points of TCM, syndrome diagnosis, tongue image, pulse condition, disease diagnosis of modern medicine, diagnosis points of modern medicine, treatment strategy, recipe medicinal, other therapeutic method, and prevention), organized into 2 dimensions (diagnosis and treatment). These dimensions and items underwent a face-to-face discussion by expert panel, to evaluate their appropriateness.

The expert panel consisted of 10 clinical experts of TCM (Dr. Ying Gao, Dr. Dachan Chen, Dr. Yanming Xie, Dr. Xuejie Han, Dr. Chuanjian Lu, Dr. Yan Huang, Dr. Xusheng Liu, Dr. Zehuai Wen, Dr. Darong Wu, and Dr. Liying Wang), 4 methodologists (Mr. Binsheng Sang, Mr. Haiyang Yu, Professor Yubo Lv, and Professor Jianping Liu). After the expert panel discussion, the structure of the instrument was developed. It contains 4 dimensions and 7 items (Table 1). The instrument was developed to assess the consistency degree between the clinical practice and TCM guideline to reflect the guideline's implementation situation. A 4-point scale was adopted to score each item (4 = strong, 3 = medium, 2 = weak, and 1 = no consistency). The higher score represents better consistency. Consistency degree with the criterion measure was judged as strong if consistency degree was >80% (the diagnosis/treatment in the medical record is consistent with the diagnosis/treatment in the guideline), medium if 60–80% (the diagnosis/treatment in the medical record is consistent with the main points of diagnosis/treatment in the guideline), weak if 40–59% (the diagnosis/treatment in the medical record is consistent with the part points of diagnosis/treatment in the guideline), and no consistency if <40% (the diagnosis/treatment in the medical record is not consistent with the diagnosis/treatment in the guideline).

### 2.2. Research Population

Medical records of patients with menopause syndrome contain the gynecologists' diagnosis and treatment practices. In our study, consistency test was conducted by comparing these medical records with menopause syndrome guideline of TCM. Two groups of medical records were extracted from the electronic health record systems of five hospitals of TCM in China (Guangdong Provincial Hospital of TCM, Hangzhou Hospital of TCM, Nanjing Hospital of TCM, Yueyang Hospital of Integrated Traditional Chinese and Western Medicine, and Zhangzhou Hospital of TCM), which were written in Appendix.

Before the extraction, all gynecologists in these five hospitals received a training class about the menopause syndrome guideline of TCM with corresponding examples of clinical cases in October 2012. After the training, one group of medical records was extracted from November 2012 to January 2013. These medical records were sample 2. In order to explore the role of the training, the other group of medical records was extracted from December 2011 to January 2012. These medical records were sample 1. The diagnosis and treatment of sample 1 and sample 2 were conducted by the same gynecologists. Each hospital was required to collect 60 medical records before and after the training, respectively.

Medical records eligible for the study were female outpatients with menopause syndrome, and those who complicated with hypertension, diabetes, and other chronic diseases, hepatitis, tuberculosis, and other infectious diseases, cardiopulmonary insufficiency, hepatic and renal insufficiency, and other diseases were excluded.

Our study was approved by local ethical committee (Ethical Committee of Guangdong Provincial Hospital of Chinese Medicine; permit number B2013-016-01).

### 2.3. Data Collection

Each hospital selected a gynecologist as the evaluation staff (Dr. Xiaoyun Wang, Guangdong Provincial Hospital of TCM; Dr. Qin Zhang, Hangzhou Hospital of TCM; Dr. Xia Chen, Nanjing Hospital of TCM; Dr. Tingting Zhang, Yueyang Hospital of Integrated Traditional Chinese and Western Medicine; Dr. Xili Huang, Zhangzhou Hospital of TCM). Suggestions for guideline implementation have been added to the instrument (Appendix). These evaluation staffs used the instrument to assess the consistency degree between medical records and menopause syndrome guideline of TCM. At the same time, suggestions for guideline implementation were also collected from them. Before the assessment, we provided the evaluation staffs with a training of how to conduct the consistency test.

### 2.4. Data Analysis

#### 2.4.1. Reliability and Validity

The data from sample 1 was used to test the reliability and validity of the instrument. Cronbach Alpha coefficient was calculated for the reliability analysis. The Cronbach Alpha coefficient of 0.7 or above was considered satisfactory [19]. Confirmatory factor analysis (CFA) was used for the construct validity analysis. We carried out CFA using maximum likelihood to test whether our factor structure fits the data. Several indices for the goodness of fit were used, including standardized root mean-squared residual (SRMR), goodness of fit index (GFI), normed fit index (NFI), Tucker-Lewis index (TLI), comparative fit index (CFI), and the adjusted goodness of fit index (AGFI). SRMR score of less than 0.08 indicates a good model fit [20, 21]. For GFI, NFI, TLI, CFI, and AGFI scores higher than 0.95 indicate a good fit to the data and scores higher than 0.90 indicate an acceptable model fit [22]. Factor loadings were classified as low (<0.30), midrange (0.30–0.59), and high (≥0.60) [23].

#### 2.4.2. The Changes of Consistency Scores between Sample 1 and Sample 2

The role of training was assessed by measuring the changes of consistency scores between sample 1 and sample 2. If the data distribution was nonnormal, the Mann–Whitney test would be applied for analysis.

#### 2.4.3. Distribution of Consistency Scores

All items were analyzed by descriptive statistics. Univariate characteristics were calculated for continuous variables. If the data came from a nonnormal distribution or the variances in each group were not equal, Kruskal-Wallis test followed by all pairwise multiple comparisons would be used for testing the specific differences among the consistency scores of 7 items in sample 1 and sample 2, respectively.

Data analysis was done using SPSS 17.0 (IBM). For the CFA, AMOS 21.0 was used. A *P* value of less than 0.05 was considered statistically significant.

## 3. Results

### 3.1. Sample Characteristics

We collected 626 medical records from 58 gynecologists in 5 hospitals. Each gynecologist provided about 11 medical records. Five consistency test data of medical records with missing data of high proportion (>30%) were deleted. Eventually, a total of 621 cases with climacteric syndrome were included in our data analysis. The average age was 48.49 years (±5.34 years). Sample 1 consisted of 300 cases (aged 44 to 66 years, mean age 49.4). Sample 2 consisted of 321 cases (aged 44 to 66 years, mean age 47.9). There was no difference among different hospitals in sample 1 and sample 2 (Table 2). Each hospital had an evaluation staff to do the consistency test before and after the training. Therefore, the assessment was conducted in a consistent manner.

### 3.2. Reliability and Validity

**Figure 1:**
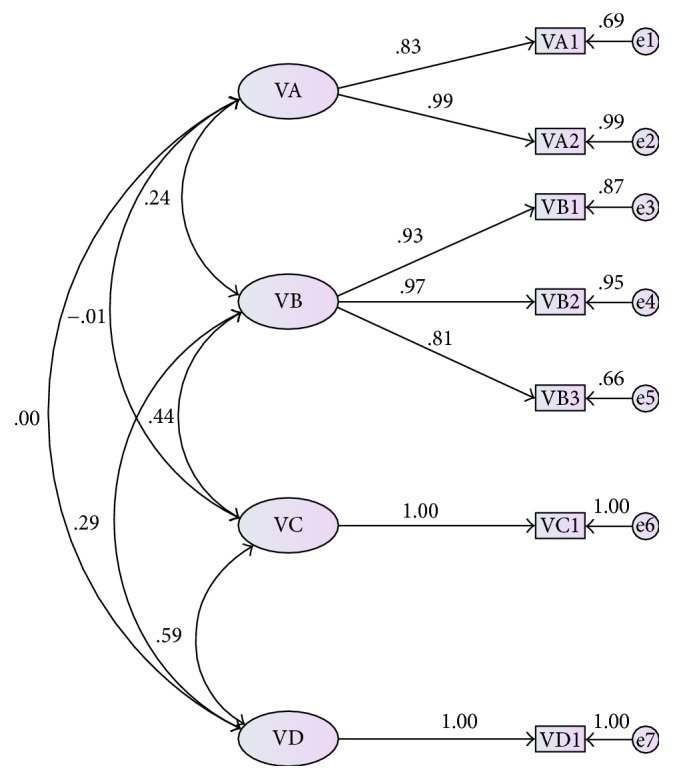
Graphical presentation of the CFA. VA: diagnosis, VA1: TCM diagnosis, and VA2: MM diagnosis; VB: syndrome differentiation and treatment, VB1: syndrome differentiation, VB2: treatment strategy, and VB3: recipe medicinal; VC = VC1: other therapeutic method; VD = VD1: prevention.

The instrument showed the internal consistency with Cronbach's Alpha coefficient of 0.73. We used CFA to check the four factorial model of the instrument. The indices for this instrument were good: *χ*^2^ = 38.01, df = 10, *P* < 0.001; SRMR = 0.04; GFI = 0.97; NFI = 0.97; TLI = 0.96; CFI = 0.98; AGFI =0.90. Except for “other therapeutic method” and “prevention,” it revealed high loadings (0.81–0.99) in general (Figure 1).

### 3.3. Changes of Consistency Scores between Sample 1 and Sample 2

Changes of consistency scores between sample 1 and sample 2 were shown in Table 3. After the normality test, data of these two samples was nonnormal (*P* < 0.0001). These changes were determined by examining the* P* values, which was generated using Mann–Whitney test. There was a significant improvement in the items of “TCM diagnosis” (*P* = 0.001), “MM diagnosis” (*P* = 0.007), “Recipe medicinal” (*P* = 0.006), and “Prevention” (*P* < 0.001) after the training.

### 3.4. Distribution of Consistency Scores

#### 3.4.1. Distribution of Consistency Scores in Sample 1

After the normality test and homogeneity test, data of 7 items in sample 1 was nonnormal (*P* values < 0.001) and heterogeneity of variance (*P* < 0.001). Then Kruskal-Wallis test was used for analysis. It revealed that there was a statistically significant difference in consistency scores among the 7 items in sample 1 (*P* < 0.001). After pairwise multiple comparisons, there was no statistically significant difference between items of “TCM diagnosis” and “MM diagnosis” (*P* = 0.602), “syndrome differentiation” and “treatment strategy” (*P* = 0.520), and “recipe medicinal” and “other therapeutic method” (*P* = 0.003), as well as “other therapeutic method” and “prevention” (*P* = 0.006). In sample 1, consistency scores of diagnosis dimension were significantly higher than the other dimensions (Table 4).

#### 3.4.2. Distribution of Consistency Scores in Sample 2

After the normality test and homogeneity test, data of 7 items in sample 2 was nonnormal (*P* values < 0.0001) and heterogeneity of variance (*P* < 0.0001). Then Kruskal-Wallis test was used for analysis. It showed that consistency scores in sample 2 had significant differences among the 7 items (*P* < 0.0001). After pairwise multiple comparisons, there was no statistically significant difference between items of “TCM diagnosis” and “MM diagnosis” (*P* = 0.787), “syndrome differentiation” and “treatment strategy” (*P* = 0.712), and “recipe medicinal” and “prevention” (*P* = 0.317). In sample 2, consistency score of diagnosis dimension was significantly higher than the other dimensions (Table 5).

### 3.5. Suggestions for Guideline Implementation

We also collected the suggestions from the evaluation staff. They mostly considered that the guideline should be published in authoritative journals. Moreover, the guideline should be provided in TCM medical institutions at all levels so as to attract feedback and promote modification during the guideline implementation.

## 4. Discussion

### 4.1. The Instrument for Measuring the Implementation Situation of TCM Guideline

Evaluating the implementation situation of guideline can help developers to make an accurate assessment on the effect of implementation. Valid and reliable instrument should thus be developed to evaluate the implementation situation. In our study, we demonstrated the reliability and validity of the instrument. We included medical records from five hospitals of different provinces in China, which enabled the collected data to be more comprehensive.

The internal consistency of the instrument was good. It indicated good reliability of the instrument in monitoring the implementation situation. Moreover, the instrument showed a coherent and theoretically consistent factor structure that the coefficients for the model fit were very good. The instrument for measuring the implementation situation of TCM guideline was developed and validated. It is suitable to be applied to indicate the implementation situation.

### 4.2. The Role of the Training in Guideline Implementation

In our study, the changes of consistency scores between sample 1 and sample 2 were used to evaluate the role of training in the guideline implementation. After the training, consistency scores of “TCM diagnosis” (*P* = 0.001), “MM diagnosis” (*P* = 0.007), and “recipe medicinal” (*P* = 0.006) were increased significantly. In the item of “other therapeutic method,” the consistency score was decreased after the training, with no statistical difference. These results indicate that the training can improve the consistency of gynecologists' diagnosis and treatment with the menopause syndrome guideline of TCM. After the training, the implementation situation is better than before. The training would promote the implementation of the guideline.

### 4.3. Implementation Situation of Menopause Syndrome Guideline of TCM

As shown in Tables 4 and 5, consistency scores of “TCM diagnosis” were similar to “MM diagnosis” in both sample 1 and sample 2, so were the “syndrome differentiation” and “treatment strategy.” In China, most clinicians of TCM usually offer health services with TCM and MM [24]. In the field of TCM diagnosis, the combination of disease and syndrome has become a basic consensus [25]. Clinicians of TCM would carry on TCM diagnosis and MM diagnosis at the same time. Therefore, the “TCM diagnosis” would have a similar consistency degree with “MM diagnosis.” Based on the TCM theory, each syndrome matches one corresponding treatment strategy. So, the “syndrome differentiation” would have a similar consistency degree with “treatment strategy.”

We also found that consistency scores of diagnosis dimension were significantly higher than the other dimensions in both sample 1 and sample 2. These dimensions of low consistency scores were “syndrome differentiation and treatment,” “other therapeutic method,” and “prevention.” It revealed that the implementation situation of treatment content in menopause syndrome guideline of TCM was not very optimistic. Generally, common reasons affecting the implementation are (1) problems of the guideline itself, such as unclear content of the guideline, backward technical content, weak operability, and lack of scientific evidence; (2) issues from guideline users, such as low awareness rate (not familiar with guideline content and related information) and low compliance (experience and habits, disapprobation, or skill requirements are difficult to achieve); (3) target disease/patient of the guideline, such as the complexity of the disease (clinical guidelines are difficult to cover all special problems), patient value, and preference; (4) environment for the guideline implementation, such as the policy system (local health care and hospital rules), facilities, and conditions. To see why these gynecologists did not use the treatment mentioned in the climacteric syndrome guideline of TCM, we looked up the questionnaire of consistency testing and found that (1) some patients with climacteric syndrome showed syndrome of kidney deficiency and blood stasis and syndrome of heart-spleen deficiency. These two syndromes and corresponding treatments are not included in the guideline. Gynecologists chose the other treatments on the basis of their own experiences. (2) Except for the main syndrome, some patients with climacteric syndrome were accompanied with other syndromes. Treatments for this situation are not mentioned in the guideline. (3) In order to improve the clinical curative effect, some gynecologists also adopted some other treatments of TCM, such as emotional therapy of TCM and auricular point sticking. So, it is considered that the main reason affecting the implementation of the treatment in the guideline was the complexity of the disease.

Although the guideline was developed by experts' consensus and its consistency score of treatment was not very optimistic, it did not mean that this guideline was not good. On the basis of the complexity of the disease, a guideline does not always cover all special problems. Evaluating the implementation situation can help us to know the room for improvement of the guideline. Therefore, we can revise the guideline on the basis of above-mentioned feedback. At present, there is a growing trend that clinical guidelines should be developed based on evidence. We suggest that the revision of the climacteric syndrome guideline of TCM can refer to the development process and methodology principles proposed by AGREE group [26], adopt systematic reviews to synthesize all the available evidence in the development of guideline, and use GRADE [27, 28] system to rate the quality of evidence and develop the grade of recommendations.

## 5. Conclusions

The instrument for measuring the implementation situation of TCM guideline is of good reliability and validity. It can help the guideline developers to measure the consistency between the actual clinical practice and the TCM guideline and reflect the implementation situation. At the same time, we can find the reasons affecting the implementation and revise the guideline. This would be conducive to making an accurate assessment on the effect of guideline implementation. This instrument is mainly used in TCM field. The method of using consistency test to measure the implementation situation of TCM guideline may provide a sample for evaluating the guideline implementation in other fields.

## Figures and Tables

**Table 1:** Consistency test model of TCM guideline.

Scale	Dimensions	Items
Consistency of guideline	Diagnosis	TCM diagnosis
		MM diagnosis
	Syndrome differentiation and treatment	Syndrome differentiation
		Treatment strategy
		Recipe medicinal
	Other therapeutic method	Other therapeutic method
	Prevention	Prevention

TCM: Traditional Chinese Medicine; MM: modern medicine.

**Table 2:** Numbers of medical records from different hospitals in sample 1 and sample 2.

Different hospitals	Numbers of gynecologists	Sample 1	Sample 2	*χ* ^2^	*P*
Guangdong Provincial Hospital of TCM	16	66	81	0.973	0.914
Hangzhou Hospital of TCM	8	57	60		
Nanjing Hospital of TCM	14	59	62		
Yueyang Hospital of Integrated Traditional Chinese and Western Medicine	8	60	60		
Zhangzhou Hospital of TCM	12	58	58		
Total	58	300	321		

TCM: Traditional Chinese Medicine.

**Table 3:** Changes of consistency scores between sample 1 and sample 2.

Dimensions	Items	Median	Sample 1				Median	Sample 2				*P*
			Frequency of each consistency degree					Frequency of each consistency degree				
			No consistency	Weak	Medium	Strong		No consistency	Weak	Medium	Strong	
VA	VA1	4.00	1	13	129	157	4.00	0	1	116	204	0.001
	VA2	5.00	0	15	117	168	4.00	0	1	111	209	0.007
VB	VB1	3.00	33	37	97	133	3.00	40	22	100	159	0.207
	VB2	3.00	27	44	110	119	3.00	38	23	109	151	0.09
	VB3	3.00	26	90	116	68	3.00	41	46	131	103	0.006
VC	VC1	3.00	40	95	113	52	2.00	52	119	87	63	0.051
VD	VC2	2.00	124	28	110	38	3.00	63	33	115	110	<0.0001

VA: diagnosis, VA1: TCM diagnosis, and VA2: MM diagnosis; VB: syndrome differentiation and treatment, VB1: syndrome differentiation, VB2: treatment strategy, and VB3: recipe medicinal; VC = VC1: other therapeutic method; VD = VD1: prevention.

**Table 4:** Distribution of consistency scores in sample 1^#^.

Dimensions	Items	Median	Frequency of each consistency degree				*P* ^a^	*P* ^b^	*P* ^c^	*P* ^d^	*P* ^e^	*P* ^f^	*P* ^g^
			No consistency	Weak	Medium	Strong							
VA	VA1	4.00	1	13	129	157	—						
	VA2	4.00	0	15	117	168	0.602	—					
VB	VB1	3.00	33	37	97	133	*∗∗*	*∗∗*	—				
	VB2	3.00	27	44	110	119	*∗∗*	*∗∗*	0.520	—			
	VB3	3.00	26	90	116	68	*∗∗*	*∗∗*	*∗∗*	*∗∗*	—		
VC	VC1	3.00	40	95	113	52	*∗∗*	*∗∗*	*∗∗*	*∗∗*	0.003	—	
VD	VD1	2.00	124	28	110	38	*∗∗*	*∗∗*	*∗∗*	*∗∗*	*∗∗*	0.006	—

VA: diagnosis, VA1: TCM diagnosis, and VA2: MM diagnosis; VB: syndrome differentiation and treatment, VB1: syndrome differentiation, VB2: treatment strategy, and VB3: recipe medicinal; VC = VC1: other therapeutic method; VD = VD1: prevention; *Notes*. ^#^Adjusted *P* value for multiple comparisons test was 0.002. *P* < 0.002 was considered to be statistically significant; ^a^*P* values of different items compared with “VA1”; ^b^*P* values of different items compared with “VA2”; ^c^*P* values of different items compared with “VB1”; ^d^*P* values of different items compared with “VB2”; ^e^*P* values of different items compared with “VB3”; ^f^*P* values of different items compared with “VC1”; ^g^*P* values of different items compared with “VD1”; ^*∗∗*^*P* < 0.001.

**Table 5:** Distribution of consistency scores in sample 2^#^.

Dimensions	Items	Median	Frequency of each consistency degree				*P* ^a^	*P* ^b^	*P* ^c^	*P* ^d^	*P* ^e^	*P* ^f^	*P* ^g^
			No consistency	Weak	Medium	Strong							
VA	VA1	4	4	0	1	116	—						
	VA2	4	4	0	1	111	0.787	—					
VB	VB1	3	3	40	22	100	*∗∗*	*∗∗*	—				
	VB2	3	3	38	23	109	*∗∗*	*∗∗*	0.712	—			
	VB3	3	3	41	46	131	*∗∗*	*∗∗*	*∗∗*	*∗∗*	—		
VC	VC1	3	2	52	119	87	*∗∗*	*∗∗*	*∗∗*	*∗∗*	*∗∗*	—	
VD	VD1	2	3	63	33	115	*∗∗*	*∗∗*	*∗∗*	*∗∗*	0.317	*∗∗*	—

VA: diagnosis, VA1: TCM diagnosis, and VA2: MM diagnosis; VB: syndrome differentiation and treatment, VB1: syndrome differentiation, VB2: treatment strategy, and VB3: recipe medicinal; VC = VC1: other therapeutic method; VD = VD1: prevention; *Notes*. ^#^Adjusted *P* value for multiple comparisons test was 0.002. *P* < 0.002 was considered to be statistically significant; ^a^*P* values of different items compared with “VA1”; ^b^*P* values of different items compared with “VA2”; ^c^*P* values of different items compared with “VB1”; ^d^*P* values of different items compared with “VB2”; ^e^*P* values of different items compared with “VB3”; ^f^*P* values of different items compared with “VC1”; ^g^*P* values of different items compared with “VD1”; ^*∗∗*^*P* < 0.001.

## Appendix

The instrument for measuring the implementation of Traditional Chinese Medicine guideline

  No. □□□□
  (1) Basic information
  (1.1) Information of evaluation staff and work unit
      Name of disease: —
      Name of guideline: —
      Name of work unit: —
      Date: —
      Name of evaluation staff: — (signature)
  (1.2) Case information
      Medical record No. —
      Gender:  Male □  Female □
      Age: □□□
      Chief complaints: —
      History of present illness: —
  (2) Diagnosis: —
  (2.1) TCM diagnosis (Consistency test)
      □ strong  □ medium  □ weak  □ no consistency
  (2.2) MM diagnosis (Consistency test)
      □ strong  □ medium  □ weak  □ no consistency
  (3) Syndrome differentiation and treatment: —
  (3.1) Syndrome differentiation (Consistency test)
      □ strong  □ medium  □ weak  □ no consistency
  (3.2) Treatment strategy (Consistency test)
      □ strong  □ medium  □ weak  □ no consistency
  (3.3) Recipe medicinal (Consistency test)
      □ strong  □ medium  □ weak  □ no consistency
  (4) Other therapeutic method
  (4.1) Other therapeutic method (Consistency test)
      □ strong  □ medium  □ weak  □ no consistency
  (5) Prevention:—
  (5.1) Prevention (Consistency test)
      □ strong  □ medium  □ weak  □ no consistency
(6) Suggestions for guideline implementation:—

*Note*. TCM: Traditional Chinese Medicine; MM: modern medicine. Consistency test: compare the information extracted from medical record with the guideline. Case information, diagnosis, syndrome differentiation and treatment, other therapeutic method, and prevention were extracted from medical record.
